# GRANDPARENT-PARENT RELATIONSHIP, CAREGIVING MEANING AND FAMILY WELLBEING: AN APIM STUDY OF 200 DYADS

**DOI:** 10.1093/geroni/igac059.3103

**Published:** 2022-12-20

**Authors:** Xu Liao

**Affiliations:** City University of Hong Kong, Hong Kong, Hong Kong

## Abstract

While many older persons contribute to their families by providing care to their grandchildren (GC), the role transition could bring challenges to both grandparents (GP) and parents (P) especially for those who evenly share caregiving duties. Despite growing research on grandparenthood, a knowledge gap remains in the study of GP-P partnership; it is essential to examine GP and P as a unit and learn how the dyadic relationship influences their family. The purpose of this study is to gain a further understanding of GP-P relationship, meaning of caregiving, and family well-being (FWB). Data from 200 GP-P dyads in China were collected by a questionnaire survey. The study participants were chosen from families with one GC aged 0–8 who is cared for by GP and P together, and identified by the GC’s teachers. The actor-partner interdependence model (APIM) was adopted in data analysis. Results showed that for co-(grand)parenting relationship and FWB, the actor effect was statistically significant for both GP and P; so was the partner effect from P to GP. Likewise, for meaning of caregiving and FWB, the actor effect was statistically significant for both GP and P; so was the partner effect from GP to P. The results advance knowledge about GP-P partnership and how feelings and actions could impact the overall family well-being. The findings can also guide the development of practices and services that enhance the function of GP-P collaboration and their family well-being.

